# Supplementary material for: Case of Mixed Infection of Toenail Caused by Candida parapsilosis and Exophiala dermatitidis and In Vitro Effectiveness of Propolis Extract on Mixed Biofilm
Source: J Fungi (Basel). 2023 May 17;9(5):581. doi: 10.3390/jof9050581 (PMC10218994; doi:10.3390/jof9050581)
Supplement: Supplementary file 1 [file jof-09-00581-s001.zip › jof-2133003-supplementary.pdf]

Journal of *Fungi*

MDPI

Table S1. Different inoculums were tested from 1.0x10³ to 1.0x10⁶ to define the best concentration.

Colony forming units									
		10^6		10^5		10^4		10^3	
		CP	ED	CP	ED	CP	ED	CP	ED
24h	106	5,79	4,71	5,41	4,69	5,41	4,74	3	0
	105	4	4,68	5,23	4,36	5,74	4,41	6,07	3,47
	104	5,78	3	5,46	3,3	3	3,3	5,69	3,47
	103	4,6	0	4,14	0	4,39	0	3,47	0
		10^6		10^5		10^4		10^3	
		CP	ED	CP	ED	CP	ED	CP	ED
48h	106	5,9	4,77	6,7	0	5,79	0	5,36	0
	105	6,3	4,39	4,44	3,3	5,86	3,6	4,77	0
	104	4,69	3,6	4,76	3,6	4,8	4,3	4,34	0
	103	4,14	3,77	0	3,3	0	0	3	0
		10^6		10^5		10^4		10^3	
		CP	ED	CP	ED	CP	ED	CP	ED
72h	106	5,69	0	4,73	0	4,77	0	4,83	0
	105	4,54	4,77	4,75	3,77	4,74	0	4,54	0
	104	5,41	4,17	5,91	4,36	6,04	0	6,65	0
	103	3,6	4,44	3	4,25	4,6	4,41	4,73	3,69
		10^6		10^5		10^4		10^3	
		CP	ED	CP	ED	CP	ED	CP	ED
96h	106	6,86	4,69	4,61	0	4,49	0	5,8	0
	105	5,43	4,81	4,51	4	4,77	0	3,9	0
	104	5,91	3,3	4,76	3	4,44	0	4,6	0
	103	0	4,32	3,3	3,69	4,17	3,3	4,39	0

CP: Candida parapsilosis
ED: Exophiala dermatitidis

Table S2. Mixed biofilm formation of the two yeasts by colony forming units.

Time	*Candida parapsilosis*	*Exophiala dermatitidis*	Mixed
24h	5,23	4,36	9,59
48h	4,44	3,30	7,74
72h	4,75	3,77	8,53
96h	4,51	4,00	8,51
